# Information Exchange Between Providers During Transitions of Surgical Care: Communication, Documentation and Sometimes Both

**Published:** 2017

**Authors:** Stacey Slager, Julie Beckstrom, Charlene Weir, Guilherme Del Fiol, Benjamin S Brooke

**Affiliations:** a University of Utah, Salt Lake City, Utah; b George E. Whalen Department of Veteran Affairs Medical Center

**Keywords:** Information, documentation, qualitative analysis, provider-provider communication

## Abstract

Poor communication of health information between healthcare providers is associated with over 80% of medical errors that occur during transitions of care [1]. We interviewed a diverse sample of primary care providers and surgical providers during their patient's transitions of care before and after surgery at a Veteran's Health Administration hospital and a large tertiary academic medical center to understand how providers communicate and exchange health information for medically complex older patient across different care settings. Our objective was to identify factors that lead to poor communication as well as strategies to optimize provider-provider communication. This paper highlights the methods providers use to communicate and document health information within two separate electronic medical record (EMR) systems during transition of care and presents a conceptual diagram of how information exchange occurs within these two EMR systems.

## 1. Introduction

The exchange of information among health care providers is an essential process that informs and affects all facets of patient care. This involves the explicit transfer of patients' medical information and responsibility for care as patients move between different health care specialty providers and settings. In all different types of health care settings, information exchange may occur as either documentation, communication or both and sometimes requires interpretation (e.g. radiology results). Documentation is any information about a patient that is contained in the patient chart and serves as a record. Health care providers write notes in the patient's chart for reasons such as recording what occurred at a clinic visit, billing purposes, and writing patient care orders for other healthcare providers to carry out. These notes are considered documentation. Communication is any interaction among provider and/or patients regarding a patient's care and treatment. Direct clinical communication such as a surgical referral note, also becomes documentation. The objective of our study is to characterize what, where, when and how surgical and primary care providers communicate clinical goals and expectations during transitions of care. Our study revealed the ways in which information is exchanged among providers within different specialties either as documentation or communication and how interchangeable these definitions can be depending on the circumstance. In this context, we define information as any data relevant to the care and treatment of a patient, regardless of how it is conveyed and who sends or receives it. This may be explicit or tacit.

## 2. Background

Transitions of care before and after surgery are critical time periods for primary care and surgical providers to communicate with each other regarding their patient's care. Provider-to-provider communication between the PCP and surgeon begins at the time of referral (if the surgery is not due to an emergency), which sometimes includes a pre-operative workup (i.e. labs, imaging, etc.). The surgeon receives the referral prior to seeing the patient at which point he/she will decide whether to order a pre-operative work-up and or conduct a procedure. Once the surgical procedure has taken place, the surgeon documents what occurred in the operating room and writes a discharge summary of the post-operative hospital course. If the surgeon and the PCP are within the same EMR system then clinical documentation can serve as communication about a patient's procedure. If these two providers are not within the same EMR system, communication and documentation become more fragmented and are not necessarily interchangeable.

The EMRs under study include the Computerized Patient Record System (CPRS) utilized at the George E. Whalen Department of Veteran Affairs Medical Center (VAMC) and Epic, a major commercial vendor system utilized by University of Utah Healthcare (UUHC). These EMR systems do not currently communicate with each other.

## 3. Methods

We prospectively identified 25 different medically complex (≥ 3 comorbidities) patients over 60 years of age referred by their PCP for major general and vascular surgery procedures. We only selected patients who were closely followed by one PCP, defined by having at least 4 recent consecutive visits or by being assigned that PCP for at least 2 years with at least 2 visits. Semi-structured interviews were conducted of the PCP and surgical providers responsible for communicating information about the patients during both the referral period and the follow-up period after surgery. Seventeen surgical providers and 16 PCPs agreed to be interviewed. Table 1 lists the demographics of the provider participants. Members from the study team went to the provider's location (clinic, hospital, or office either at the VA medical center or at a University facility) for a brief interview that lasted on average 10 minutes. The interviews were audio recorded using the interviewer's smart phone, transcribed, and coded using Atlas.ti (Berlin) qualitative analysis software. Codes were developed first by categorization based on the questions asked, then by Grounded Theory [2], in which themes emerge by salience and frequency. The team met frequently to agree on a final code book then two coders (SS and JB) worked to achieve inter-rater reliability. Transcripts were de-identified. All interviewees signed an informed consent document.

The questions the providers were asked centered on the patient's episode of care regarding their pre- and post-operative functional, social and cognitive status, the goals of the patient, PCP and surgeon (and what the interviewee thought the other's goals were), as well as communication preferences and expectations based on assumptions one provider had about the other's information needs. It is this last aspect that is the focus of this presentation. Table 2 lists the questions we asked about communication and documentation.

## 4. Results

Based on our conversations with providers, it became clear that clinical communication and documentation were employed at different times and for different reasons. When there was no urgent need for a surgeon to communicate with a PCP, a standard visit note was sufficient to document the patient's status and clinical course, particularly if no immediate follow up was required. For example, a trauma surgeon only communicates if there is a problem:

I don't personally talk to primary care physicians after surgery. The only form of communication that I would have is through Epic if they're (the PCP) on Epic and if they're going to a skilled nursing facility or residence, we'll do a transfer phone call. So there would be discussion at that level if there was a very specific issue where we needed information or we needed to convey information to the PCP we would make a phone call.

The reasons for documenting versus communicating in clinical settings are not always the same. Provider-provider communication is usually about the current situation or near future (including follow up requirements), and usually regarding patient care. One provider noted the bureaucratic requirements of documentation:

I think our healthcare environment is really complicated so the more communication we can have the better but I also think that sometimes adding this - enforcing communication … that doesn't help either like you are just adding some other bureaucratic thing to do.

A heuristic mentioned by several providers was whether or not the patient experienced post-surgical complications. If there were no complications, many were content to rely upon conveying the patient's surgical course to the other provider via documentation alone. If the patient experienced complications, many providers would attempt to contact the other directly:

If all goes according to plan the PCP would receive a copy of my operative note and a copy of the discharge as long everything was smooth if there is any complications the PCP gets a phone call.

Workflow constraints of clinical documentation and communication caused some concern as well. Information does not arrive in time for decision making. The lack of consistency with what comes in a referral or post-operative note was mentioned by several providers, and lack of information required more effort to determine what was missing. One surgeon noted:

I mean, it varies. Referrals sometimes come with lots of information, sometimes with no information at all.

## 5. Conceptual Diagram

**Figure 1:**
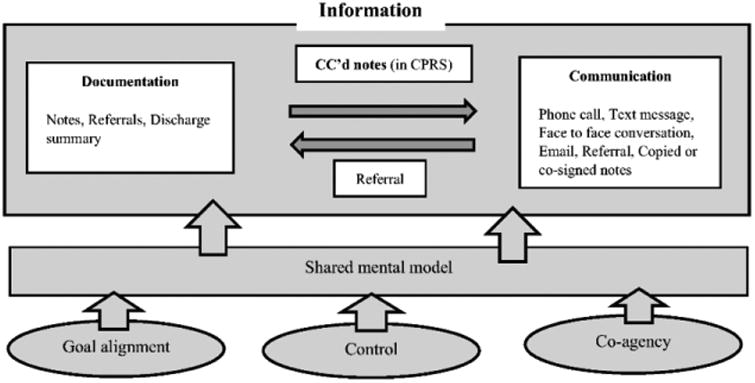
Conceptual Diagram of information exchange within the shared communication/documentation space during transitions of surgical care

The information space used by surgeons and PCPs can be viewed as a joint cognitive system (JCS), where communication and sharing of clinical goals can be measured by goal alignment, control and co-agency [4]. Based on responses of providers in our study regarding information flow, we modified this existing conceptual framework to incorporate clinical documentation and communication within a shared information space (Figure 1). Documentation that gets shared with others and communication help to establish a shared mental model. [5] Documentation includes clinical notes, referrals and discharge summaries (for our purposes here). Communication includes phone calls, cell phone text messages, in-person conversations, emails, referrals, and, with CPRS, copied or forwarded clinical notes for co-signing. Clinical notes are included both as documentation since they remain in the patient's record and communication since the first provider adds the second provider as a way to convey the information contained within it. Conversely, a referral is a means of communicating clinical information, in this case, a patient with a need for surgery, and that referral document ends up in the patient's record. The aim of information sharing is goal alignment, where both parties desire the same outcome for the patient.

## 6. Discussion

All of health care functions within a shared information space. Treatment cannot be given without information about the patient's chief complaint or condition. Information is conveyed whether by communication or documentation, depending on the urgency, patient's post-surgical complication status, and the information that needs to be conveyed.

Invariably, information exchange between different providers is prone to error and important data about a patient's care will fall through the cracks. PCPs do their best to communicate information before surgery that will allow surgeons to know whether patients are good candidates for surgery. However, a lot of contextual information about a given patient's medical history is missing. Similarly, surgeons communicate to PCPs about whether a patient's surgical procedure was successful with no postoperative complications, but it is difficult to know whether that information is received and understood. Some of the providers we spoke to were perfectly happy with the current EMR system as a means for conveying patient information, while others wished they had the time to communicate personally to the other provider about a patient's status. Both provider types want to prevent gaps in information exchange during transitions of care and ensure patients emerge from the surgical episode free from adverse events.

### 6.1 Limitations

Some of the participants mentioned the challenges when they are dealing with providers who work within a different EMR system, and that was not discussed here. Different provider types have different information needs; that will be discussed in a separate paper.

## 7. Conclusion

It is critical for all healthcare providers to have readily available information about a patient's care and status during transitions of surgical care. More research is needed to determine information needs of other provider types so that EMR systems and applications can be designed to facilitate optimal information exchange.

## Figures and Tables

**Table 1:** Demographics of PCPs and surgical providers

Variable	n/31, %	short description
UUHC	19, 61%	University of Utah Health Care System
VA	12, 39%	Veteran's Health Administration
Female	14, 45%	
Male	17, 55%	
PCP	15, 48%	primary care provider
Surgeon – (Vascular)	10, 63%	vascular surgeon
Surgeon – (other)	6, 37%	general or other surgeon

**Table 2:** Interview questions concerning communication and documentation

For PCPs What kind of information do you think the surgeon needed to know? How do you normally communicate with surgeons? What information would you like to receive back after the procedure?
For Surgeons What information would you want from the PCP to make a decision about this patient? What additional information needs to be communicated to the primary care provider after surgery about this patient? How do you normally communicate with PCPs?

## References

[1] Solet DJ, Norvell JM, Rutan GH, Frankel RM (2005). Lost in translation: challenges and opportunities in physician-to-physician communication during patient handoffs. Acad Med.

[2] Strauss A, Juliet C, Denzin N, Lincoln Y (1994). Grounded theory methodology: An overview. Handbook of Qualitative Research 1^st^ ed.

[3] Weir CR, Nebeker JJ, Hicken BL, Campo R, Drews F, Lebar B (2007). A cognitive task analysis of information management strategies in a computerized provider order entry environment. J Am Med Inform Assoc.

[4] Blomberg O (2011). Conceptions of Cognition for Cognitive Engineering. Int J Aviat Psychol.

[5] Jonker CM, van Riemsdijk MB, Vermeuelen B (2010). Shared Mental Models: A conceptual analysis. Coordination, Organizations, Institutions, and Norms in Agent Systems VI.

